# Blood flow shapes intravascular pillar geometry in the chick chorioallantoic membrane

**DOI:** 10.1186/2040-2384-2-11

**Published:** 2010-07-07

**Authors:** Grace S Lee, Nenad Filipovic, Lino F Miele, Miao Lin, Dinee C Simpson, Barry Giney, Moritz A Konerding, Akira Tsuda, Steven J Mentzer

**Affiliations:** 1 Laboratory of Adaptive and Regenerative Biology, Brigham & Women's Hospital, Harvard Medical School, Boston MA, USA; 2 Molecular and Integrative Physiological Sciences, Harvard School of Public Health, Boston, MA, USA; 3 Institute of Functional and Clinical Anatomy, University Medical Center of the Johannes Gutenberg-University Mainz, Germany; 4 Faculty of Mechanical Engineering, University of Kragujevac, Serbia

## Abstract

The relative contribution of blood flow to vessel structure remains a fundamental question in biology. To define the influence of intravascular flow fields, we studied tissue islands--here defined as intravascular pillars--in the chick chorioallantoic membrane. Pillars comprised 0.02 to 0.5% of the vascular system in 2-dimensional projection and were predominantly observed at vessel bifurcations. The bifurcation angle was generally inversely related to the length of the pillar (R = -0.47, P < .001). The pillar orientation closely mirrored the axis of the dominant vessel with an average variance of 5.62 ± 6.96 degrees (p = .02). In contrast, the variance of pillar orientation relative to nondominant vessels was 36.78 ± 21.33 degrees (p > .05). 3-dimensional computational flow simulations indicated that the intravascular pillars were located in regions of low shear stress. Both wide-angle and acute-angle models mapped the pillars to regions with shear less than 1 dyn/cm^2^. Further, flow modeling indicated that the pillars were spatially constrained by regions of higher wall shear stress. Finally, the shear maps indicated that the development of new pillars was limited to regions of low shear stress. We conclude that mechanical forces produced by blood flow have both a limiting and permissive influence on pillar development in the chick chorioallantoic membrane.

## Introduction

The mechanical influence of blood flow on vessel structure is a fundamental question in developmental [1] and adaptive [2] biology. In the chick chorioallantoic membrane, a common model of microvascular network development, the extra-embryonic area undergoes limited development in the absence of blood flow [3,4]. In later embryogenesis, the onset of a heartbeat and active blood flow is associated with dramatic changes in both embryonic and extra-embryonic vessels [5-7]. In humans, physiological conditions such as growth and exercise lead to adjustments in the structural properties of the vascular network [8]. In pathologic conditions such as inflammation [9,10] and ischemia [11,12], structural adaptations appear to be essential for tissue repair and regeneration. Despite these convincing network-level observations, there is little in vivo data on the local interaction between blood flow and vessel structure.

Attempts to clarify the mechanical influence of flow on blood vessels have focused on endothelial cell responses to varying flow patterns in vitro. Flow chamber studies have demonstrated that mechanical forces, such as wall shear stress, have a profound effect on gene transcriptional activity [13-15] and endothelial phenotype [16-18]. In vitro umbilical vein endothelial cells exposed to laminar shear stress reorganize and elongate their cytoskeletal axes in the direction of flow [19]. The response of cultured endothelial cells to in vitro shear stress can also include lamellipodial protrusion and mechanotaxis in the direction of flow [20,21]. The translation of these in vitro endothelial cell observations to in vivo structural change is less clear.

To define the local influence of intravascular flow fields on vessel structure, we have studied 2^nd ^and 3^rd ^order extra-embryonic microvessels in the chick chorioallantoic membrane (CAM). The 2^nd ^and 3^rd ^order CAM vessels are part of an experimentally accessible planar network that, in contrast to the complex gas exchange and nutrient function of the CAM capillaries, has a simple transport function. More importantly, the 2^nd ^and 3^rd ^order CAM vessels have a unique morphologic feature; namely, intravascular tissue islands or "pillars" [22]. Discrete structures within the blood stream, pillars have several potential advantages in evaluating the effect of blood flow on local vessel development: 1) pillars are lined with normal-appearing endothelium [23] suggesting a normal responsiveness to intraluminal flow fields, 2) pillars are discrete structures indicating that local changes in pillar geometry have a minimal effect on global blood flow, and 3) pillars can be identified by time-series intravital 2D imaging providing a simultaneous assessment of pillar geometry and surrounding blood flow.

In this report, we used geometry and blood flow measurements derived from intravital microscopy imaging to map the mechanical forces within the CAM vessels--including wall shear stress and blood pressure--using 3D computational flow simulations. Pillar geometry suggested the spatial constraint of high wall shear stress. Further, the development of new pillars was limited to regions with low shear stress. The result suggests both a limiting and permissive influence of wall shear stress on pillar development in the CAM.

## Methods

### Eggs

Specific pathogen-free, fertilized White Leghorn chicken eggs (G. gallus *domesticus*) were obtained from Charles River Laboratories (Franklin, CT). The care of the animals was consistent with guidelines of the American Association for Accreditation of Laboratory Animal Care (Bethesda, MD).

### Ex ovo culture

For all experiments, a modified, ex ovo (shell-less) culture method was used [24]. Briefly, the eggs were kept in an R-COM 20 digital incubator (GimHae, Korea) at 37.5°C and 70% humidity with automatic turning for 3 days. On embryonic development day (EDD) 3, the eggs were sprayed with 70% ethanol, air-dried in a laminar flow hood and explanted into a 20 × 100 mm Petri dish (Falcon, BD Biosciences, San Jose, CA). The ex ovo cultures were maintained in a humidified 2% CO_2 _incubator at 37.5°C. To optimize the selective examination of the 2^nd ^and 3^rd ^order conducting, as well as facilitate intravital microscopy identification of the intravascular pillars, intravital microscopy was performed on EDD 13-16.

### Intravital microscopy system

The CAM was imaged using a Nikon Eclipse TE2000 inverted epifluorescence microscope using Nikon Plan Apo 10x and Plan Fluor 20x objectives. The microscope was custom-fitted with an insulated 37°C convective warming unit with moderate relative humidity [25]. An X-Cite (EXFO, Vanier, Canada) 120 watt metal halide light source and a liquid light guide were used to illuminate the CAM. Excitation and emission filters (Chroma, Rockingham, VT) in separate LEP motorized filter wheels were controlled by a MAC5000 controller (Ludl) and MetaMorph software 7.5 (Molecular Devices, Downingtown, PA). The 14-bit fluorescent images were digitally recorded with an electron multiplier CCD (EMCCD) camera (C9100-02, Hamamatsu, Japan). Images were routinely obtained at frame rates exceeding 50 fps with 2 × 2 binning. The images were recorded in image stacks comprising 100 to 500 frames of video sequences on a Dell Precision workstation (3.06 Ghz dual Xeon processors, 15,000 rpm ultra-SCSI hard drives, 4 gb RAM and an Nvidia Quadro 3450 graphics card with 512 mb memory). The CAMs at EDD13 thru EDD16 were imaged with intermittent time-lapse videos over 6 to 24 hour time period. Selection of vessels was based on an initial visual survey; identified intravascular pillars were then studied in detail. There was no attempt at uniform or random sampling.

### Fluorescent tracers

The fluorescent plasma marker used for intravital imaging was a 5% fluorescein isothiocyanate (FITC)-dextran (2,000,000 MW; Sigma-Aldrich, St. Louis MO) solution prepared in normal saline immediately prior to injection. In some intravital microscopy experiments, green fluorescent (ex 430 nm; em 510), neutrally-charged, polystyrene spheres (10^8 ^beads/ml) were injected with the plasma marker [26]. The 0.5 um microspheres were labeled with derivatives of the BODIPY fluorochrome (ex 488 nm, em 510 nm) using organic solvents (Invitrogen, Eugene, OR). The plasma marker and intravascular tracer solution were injected into the CAM circulation using a micro-fine 0.3 ml insulin syringe with a 30G needle (BD, Franklin Lakes, NJ).

### Image analysis

Analysis of video images was performed with MetaMorph (Molecular Devices). Image stacks were created from the 100 to 500 frame sequences. The image stacks were processed with standard MetaMorph filters. After routine thresholding, the image sequences were measured using MetaMorph's integrated morphometry applications. Morphometric measurements such as area, length, orientation, perimeter, hole area and prolate volume were routinely obtained.

### Time-series flow visualization

The stream-acquired images were stacked to create a time series of 100 or 500 consecutive frames. The stacks were systematically analyzed to ensure the absence of motion artifact. The stack "maximum" operation selected the highest intensity value for each pixel location throughout the time series. Conversely, the stack "minimum" operation selected the lowest intensity value for each pixel location. Other filters such as the "median" operation were similarly applied. The resultant image produced a time series reconstruction of the vessel during the time interval of the image stack.

### Time-series analysis

**Figure 1:**
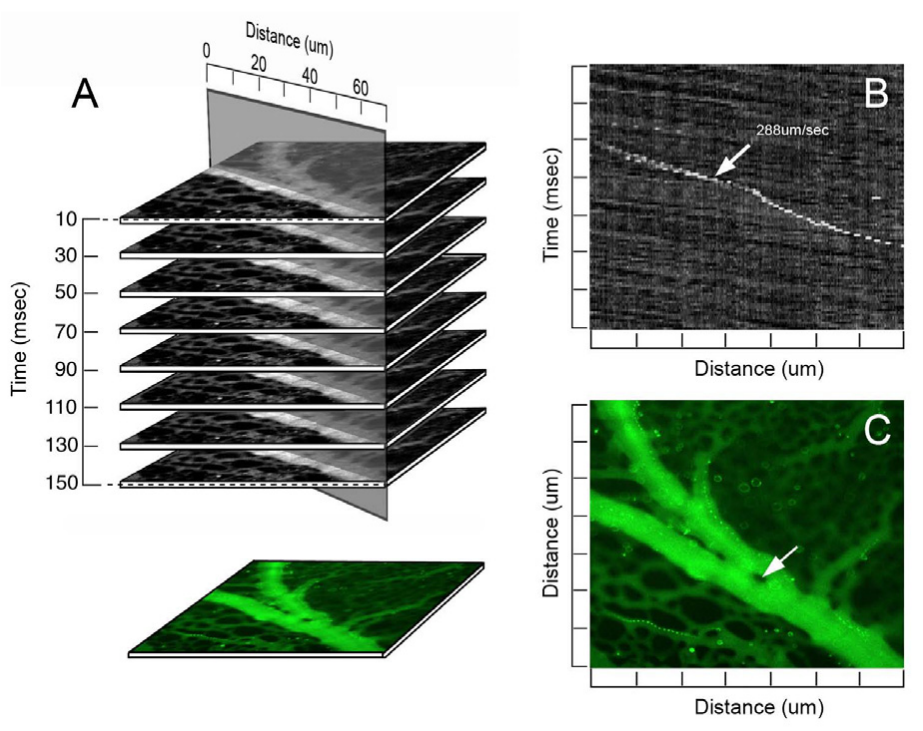
**The digital images were acquired at a single wavelength (ex 430 nm; em 510 nm). **The recorded image stacks were analyzed for flow velocity (B) and recombined into a composite time-series image (C). A,B) A line of selectable orientation and width was drawn along the vessel axis. The distance-time plane (B) provided a longitudinal view over the selected length of the vessel. Cells or particles were tracked through multiple planes of the stack permitting a visual correlation in each plane. The white object (arrow) represents a fluorescent particle; the slope of the diagonal line represents the velocity of the particle in the flow stream. Note the different slope of the background speckle pattern--an observation suggesting the particle is near to, or interacting with, the vessel wall. A,C) The image stack was digitally recombined and pseudocolored for presentation as a time-series image (C). The region within the vessel demonstrating no detectable fluorescence (arrow) was defined as an intravascular pillar.

Flow patterns were demonstrated using time-series plots created by measuring the intensity values of a region of interest through a time-based series of images. The time-series plots were constructed using the MetaMorph kymograph application [27]. The application was used to create a cross-sectional view of grayscale intensity values along a region or "transept" drawn on the image stack (Figure 1)[28]. The average pixel intensity across the vessel width was used to track movement in the time-series. The image stack was preprocessed with minimal background subtraction to improve both the signal-to-noise ratio and the sensitivity of motion detection. The resulting kymograph image was analyzed with a line tool to define the vessel segment distance, as well as the time and velocity data for the region. By convention, the transept distance was plotted on the X-axis and the descending time-series was plotted on the Y-axis. The designation of dominant and nondominant vessels was determined by volumetric flow calculations at the bifurcation.

### Image stitching

Video mapping was performed by the serial acquisition of 3 × 3 image stacks. The image stacks were acquired using MetaMorph to control of an LEP XY motorized stage (Ludl); stage accuracy and computer control minimized the time interval between image stacks (<2 sec). After the image stacks at the grid positions were acquired, the images were stitched together for a panoramic video, using the MetaMorph stack montage application.

### Measurement of vessel-pillar angles

Similar to other branch angle studies emphasizing localized geometry [29], the method of branch angle morphometry was designed to be sensitive to variation at the apex of the bifurcation. In layered images, maximally-sized spheres were inscribed in each vessel at the bifurcation. Sequential spheres within each vessel were inscribed so that the surface of the sphere intersected the centerpoint of the preceding sphere. The centerline track of the first two spheres was used to define the vessel coordinates. After routine calibration and thresholding, the pillar axis was determined by the MetaMorph integrated morphometry application. The bifurcation angle was measured as the angle between the pillar and vessel axis.

### Finite element mesh (FEM) of vessel bifurcation

To develop a finite element mesh of the vessel bifurcation, models were constructed with and without the pillar. The construction of 3D FEM for non-symmetrical, irregularly shaped 3D bifurcating vessel was based on geometry derived from intravital microscopy. Further, the transition at the pillar-wall interface required curved distortion of the mesh to match the morphology of the images from intravital microscopy [30]. Otherwise, geometric measurements provided by intravital microscopy and digital image analysis were represented as faithfully as possible in the FEM model. As automation of the FEM generation was not possible at this stage, a customized approach was required for each model. Segmentation was performed on the original 2D intravital microscopy image to obtain 2D polylines of the countours. Subsequently, 2D splines were constructed from the polylines. Finally, a 3D FEM model made of 6 blocks for smooth mesh continuity was created using 3D NURBS (Non-Uniform Rational B-Spline) [31]. In addition, two blocks of finer mesh were used around the pillar region to detect subtle changes in wall shear stress.

### Flow solver

The modeling was performed using a continuum approach. Governed by the Navier- Stokes equations and the continuity equation, the three-dimensional flow of a viscous incompressible fluid was expressed:(1)(2)

where  was the blood velocity in three different directions v_x_,v_y_, and v_z_, *ρ *was the fluid density (1.05 g/cm^3^), and *p *was pressure, *μ *was the dynamic viscosity (0.03675 g/cm/sec). Equation (1) represented the balance of linear momentum, while equation (2) expressed the incompressibility condition. The code was validated using the analytical solution for shear stress and velocities through a straight expanding tube [32].

### Boundary conditions

A parabolic velocity profile was prescribed for all calculations at the inlet of the proximal vessel; the inlet was more than 10 diameters away from the bifurcation region. A zero free traction boundary condition was kept at the outlet sections of distal limbs. This assumption was based on a steady flow condition and the parabolic velocity profile at the inlet and outlet cross-section. Reynolds number (Re) was defined as(3)

where  was the mean velocity of the prescribed inlet parabolic velocity profile, and the vessel diameter *D *was measured by intravital microscopy. Resulting Re was always less than 1.0, suggesting that the blood flow modeled here was in a viscous flow regime [33].

### Wall shear stress

The wall shear stress is calculated as , where  denotes the tangential velocity immediately to the walls, and  is the normal direction at the vessel wall. We first calculate the tangential velocity at the integration points near the wall surface, and then numerically evaluate the velocity gradient ; finally, we obtained 3 components (τ_*x*_, τ_*y*_, τ_*z*_,) of the wall shear stress vector  by multiple the velocity gradient by the viscosity coefficient *μ*. The effective value of the wall shear stress τ_*eff *_at the finite element mesh nodes on the surface was calculated as .

### Computational flow dynamics

The numerical model was developed using custom code using the C++ object oriented programming language and OpenGL graphic library [32,34-36]. The brick finite elements with 8 nodes used for our numerical simulation was more efficient than 4 node linear tetrahedral elements [37]. The code was validated using the analytical solution for shear stress and velocities through a straight tube [32,35]. The system of equations (1) was nonlinear due to the convective term and an unsymmetric Gaussian solver was implemented. An eight node finite element was employed with eight unknown velocities and constant pressure over the element which was recovered in the postprocessing calculation. Mesh independence was reached at 70,000 to 80,000 finite elements with error less then 0.1% for shear stress distribution. A smooth boundary surface was created to maintain a high resolution finite element mesh. For each calculation`, a parallel version of the solver [38] required 2 hours on 10 parallel processors with 2 GB RAM.

### Statistical analysis

Significance estimates were based on multiple comparisons of paired data by Student-Newman-Keuls or Mann-Whitney test for non-parametric analysis of variance. The values for vessel and pillar orientation for each bifurcation were exported from MetaMorph and plotted in Excel 2007 (Microsoft, Redmond WA). Pearson correlations to the unamplified control were determined using Systat 12 statistical software (Chicago, IL). The significance level for the sample distribution was defined as P < .05.

## Results

### Spatial distribution of pillars

**Figure 2:**
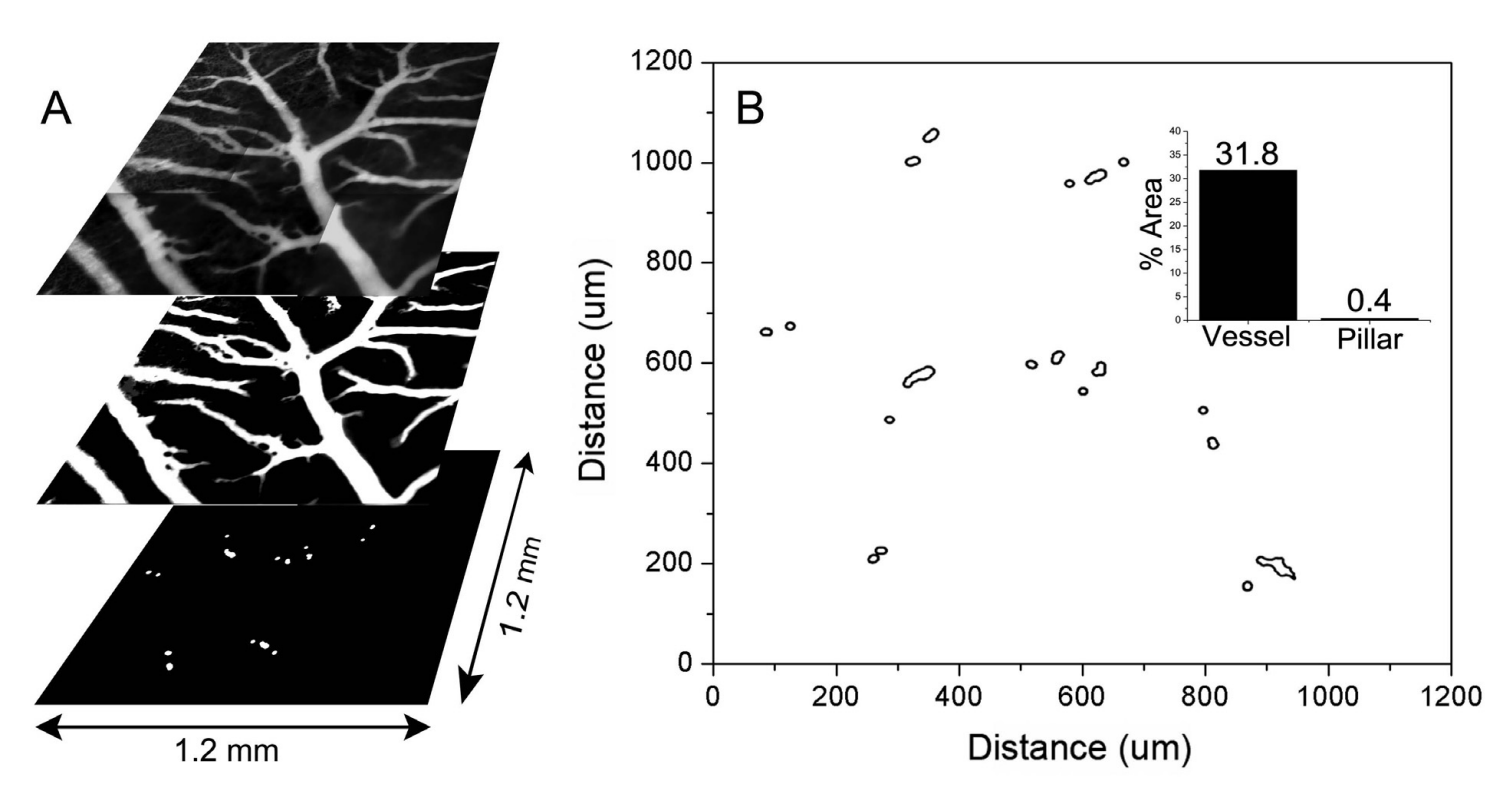
**Spatial distribution of pillars in a region of the CAM. **Time-series images of 9 contiguous regions of a CAM, previously injected with FITC-dextran, were digitally reconstructed and stitched into a 3 × 3 montage (A). The vessels were thresholded, binarized and mapped to a 2D grid (B). Morphometric analysis of the binarized image provided a relative measure of both vessel and pillar area. The vessels comprised 31.8% of the total surface area of the CAM in 2D projection; the intravascular pillars comprised 0.4% of the vessel area (inset).

The spatial distribution of pillars in the maturing CAM, EDD 13-16, was assessed by intravital microscopy. Using digitally recombined time-series image stacks (>100 images), intravascular pillars were defined as intraluminal structures with no detectable plasma marker fluorescence. Mapping of the CAM microcirculation demonstrated that the pillars had varied shapes and were distributed over multiple generations of the vascular tree (Figure 2). The majority of pillars (83%) were located within one vessel diameter of a bifurcation. Based on spatial maps of 64.2 mm^2 ^of CAM in 41 different ex ovo cultures, the pillars comprised 0.02 to 0.5% of the 2^nd ^and 3^rd ^order conducting vessels in 2-dimensional projection.

### Blood flow and pillar geometry

**Figure 3:**
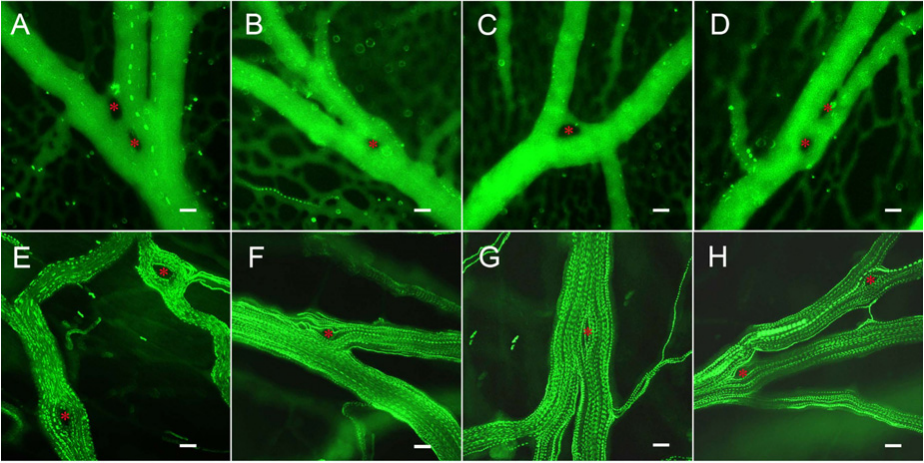
**Time-series flow visualization of the CAM intravascular pillars using fluorescence intravital videomicroscopy and intravascular tracers. **A-D) CAM vessels visualized with the plasma marker FITC-dextran and low-density fluorescent particle tracers. E-H) CAM vessels visualized with high-density particle tracers. Pillars (red asterisk) were identified as intravascular areas with no plasma marker or particle tracer throughout the time-series. Bar = 20 um.

**Figure 4:**
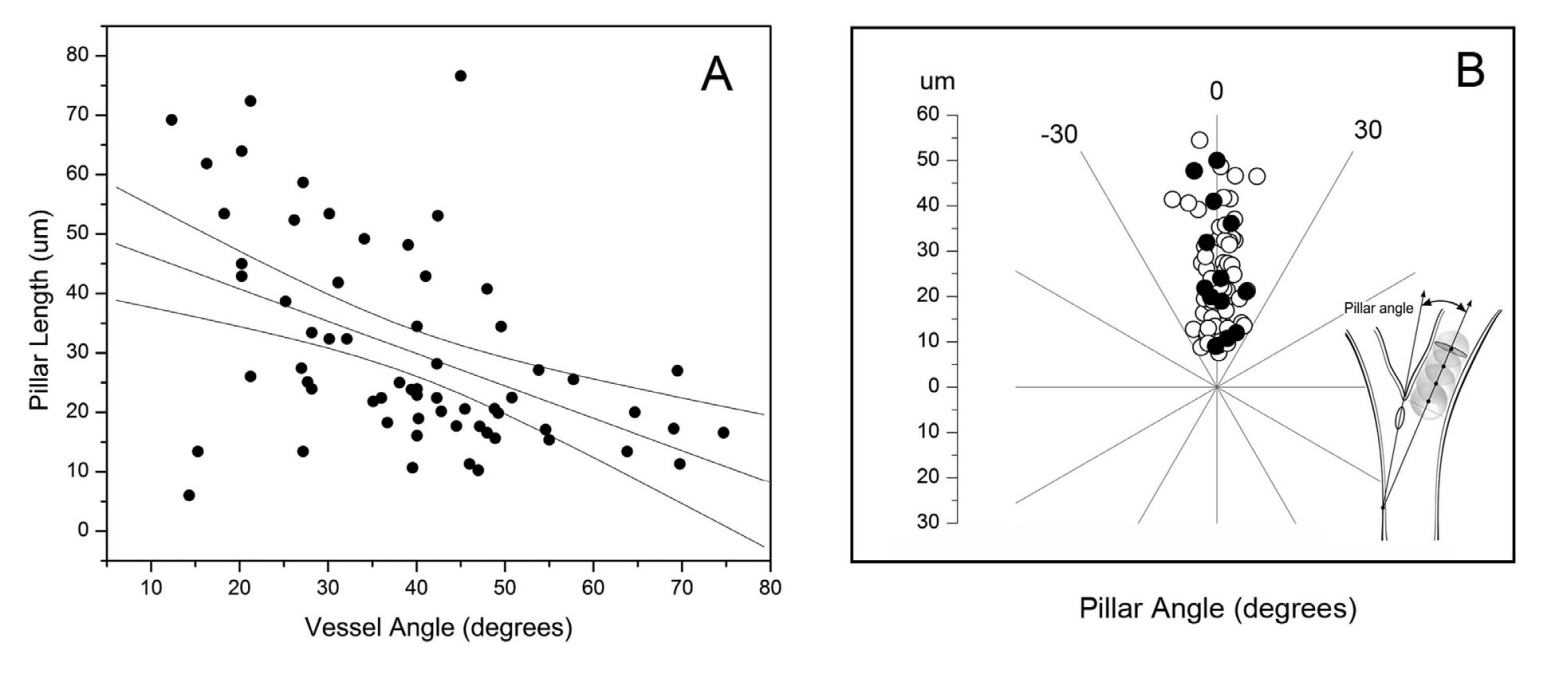
**Spatial plots of intravascular pillar anisotropy. **A) Morphometry of intravascular pillars at vessels bifurcations demonstrated an inverse correlation between pillar length and vessel angle (R2 = 0.28). B) Comparison of the orientation of the pillar axis and vessel axis. Morphometry determined the longitudinal axis and length of the pillar relative to the vessel axis (arbitrarily assigned 0 degrees). Bifurcations reflecting convergent (open circle) and divergent (closed circle) flow are shown. Regression coefficients reflected an adjusted R2 = 0.05; p = .02.

The relationship of pillar geometry to intravascular blood flow was analyzed by time-series intravital microscopy (Figure 3). Visual inspection of the pillar shape suggested that 1) narrow angle bifurcations were associated with elongated pillars, and 2) pillars were consistently oriented parallel to the dominant vessel streamline. To quantify the relationship between vessel angle and pillar length, morphometric analysis of 84 bifurcations was performed. The bifurcation angle was generally inversely related to the length of the pillar suggesting a relationship with intraluminal blood flow (R = -0.47, P < .001; Figure 4A). To analyze this relationship in detail, the pillar axis relative to the bifurcating vessels was examined by digital morphometry. The axis of the dominant vessel at a bifurcation was arbitrarily assigned an orientation of 0 degrees. When the axis of the pillar was determined by morphometry, the pillar orientation closely mirrored the axis of the dominant vessel with an average variance of 5.62 ± 6.96 degrees (p = .02; Figure 4B). In contrast, the variance of the pillar orientation with the nondominant vessels was 36.78 ± 21.33 degrees (p > .05). Further, there was a trend for the longer pillars to more closely reflect the orientation of the flow stream (Figure 4B).

### Mechanical forces and pillar shape

**Figure 5:**
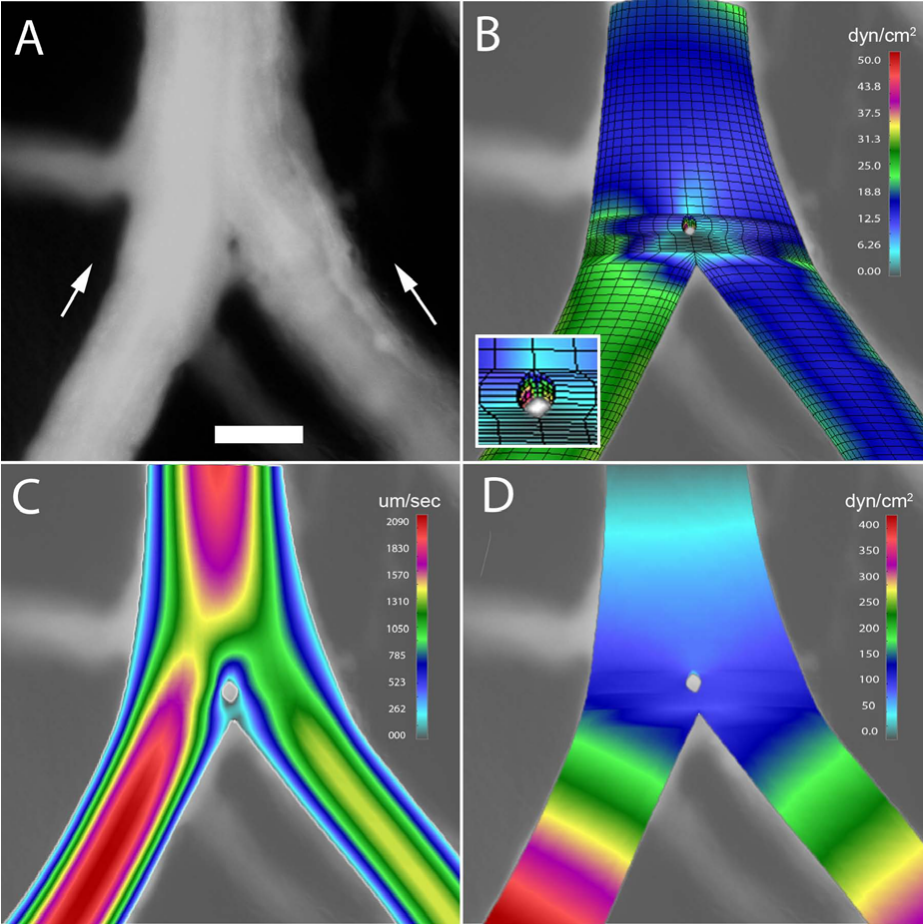
**3D computational flow modeling of a wide angle bifurcation (60 degrees) in the CAM. **A 3D finite element model was constructed based on geometry obtained from intravital microscopy; computational flow dynamics was calculated based on measured intravascular flow velocity. A) A digital recombination of a 100 consecutive images demonstrating an intravascular pillar near the bifurcation of a converging flow stream; B) wall shear stress, C) blood velocity, and C) blood pressure are also shown. Bar = 50 um

**Figure 6:**
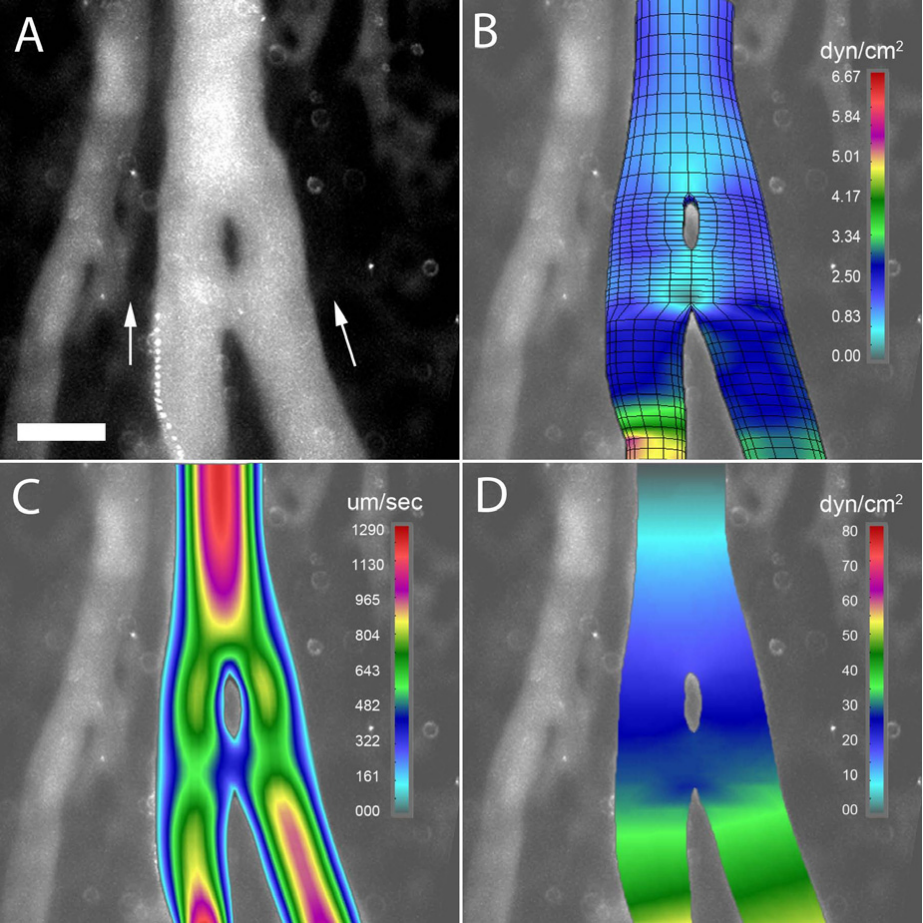
**3D computational flow modeling of an acute angle bifurcation (5 degrees) in the CAM. **A 3D finite element model was constructed based on geometry obtained from intravital microscopy; computational flow dynamics was calculated based on measured intravascular flow velocity. A) A digital recombination of a 100 consecutive images demonstrating an intravascular pillar near the bifurcation of a converging flow stream; B) wall shear stress, C) blood velocity, and C) blood pressure are also shown. Bar = 50 um.

The mechanical forces potentially shaping pillar geometry were studied by 3D computational flow modeling; convergent flow models were constructed to reflect the flow conditions observed in 78% (78/101) of bifurcations containing a pillar. In the wide-angle flow models (Figure 5), a region of low shear stress--less than 0.5 dyn/cm^2^--was observed near the bifurcation (Figure 5B). This region of low shear stress persisted in models without the pillar (not shown). Neighboring these low shear stress regions were areas of higher shear stress indicating that the pillar was constrained, and potentially shaped, by these lateral forces (Figure 5B, inset). In the acute-angle flow models (Figure 6), a relatively low shear area--less than 0.8 dyn/cm^2^--was demonstrated both upstream and downstream from the bifurcation (Figure 6B). Similar to the wide-angle models, the regions of low shear stress persisted in models without an intravascular pillar (not shown). In contrast to wall shear stress, the distributions of flow velocity and pressure were not spatially related to pillar geometry (Figure 5C, D; Figure 6C, D).

### Mechanical forces and pillar induction

**Figure 7:**
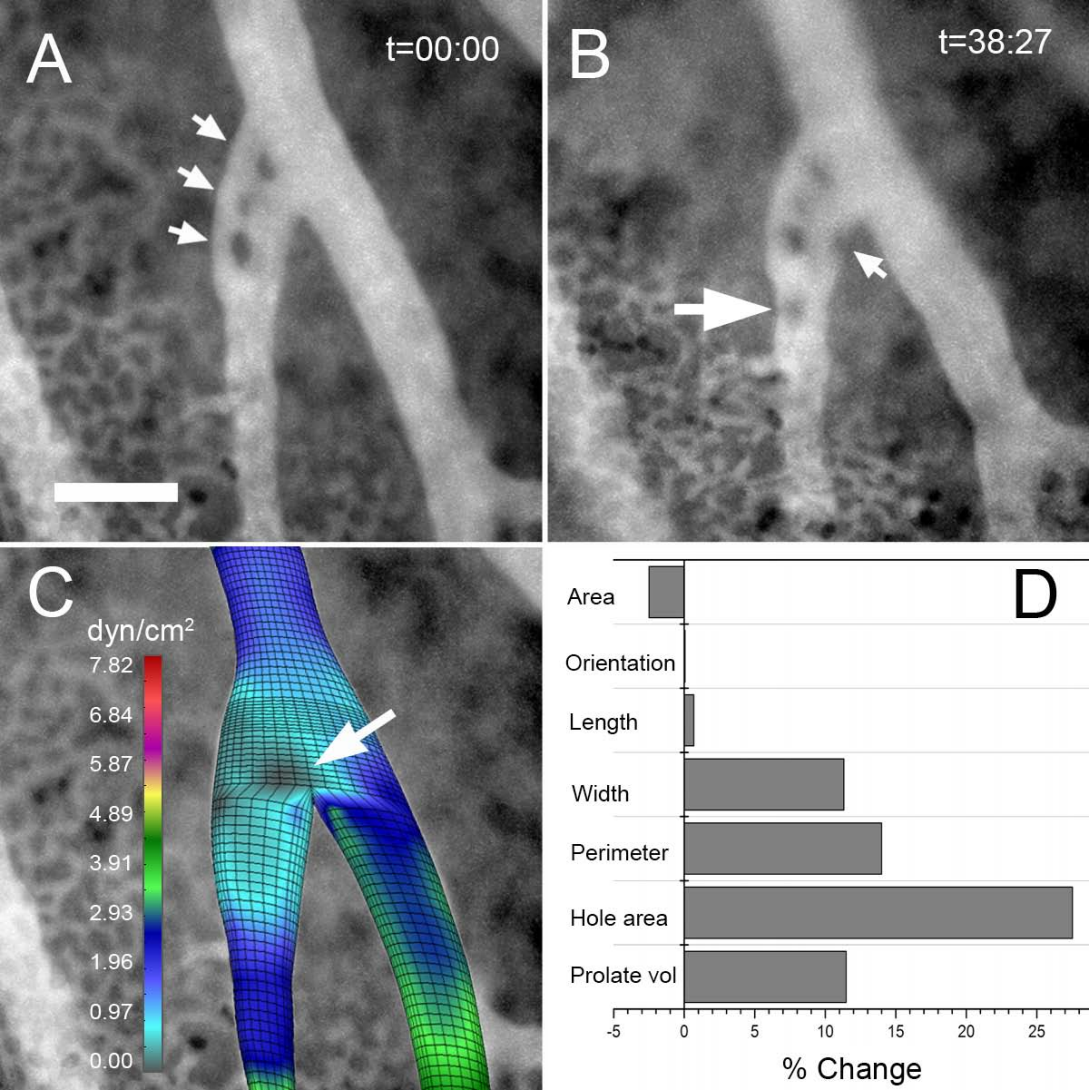
**Time-series visualization of a developing intravascular pillar using fluorescence intravital videomicroscopy. **Image stacks (100 images) of the CAM vessels plasma marker FITC-dextran were obtained at t = 0 and t = 38 minutes. A) The initial image stack revealed 3 intravascular pillars (arrows). B) At t = 38 minutes, a new intravascular pillar was identified (large arrow) accompanied by notable membrane irregularity (small arrow). C) Computational flow analysis of the vessel bifurcation without pillars demonstrated an area of low shear stress at the site of the initial pillar (arrow). D) Morphometry of the initial (A) and subsequent (B) vessels showed a significant increase in pillar area (hole area) and membrane irregularity (perimeter). Bar = 50 um.

The effect of mechanical forces in shaping pillar geometry suggested a potential role in initiating pillar formation. Most pillars, particularly at vessel bifurcations, demonstrated only small changes in pillar geometry during culture. Occasionally, rapid development of intravascular pillars was observed; these pillars typically formed downstream of pre-existing pillars (Figure 7A, B). Flow modeling indicated that new pillars formed in regions of relatively low shear stress (Figure 7C)--a finding suggesting a permissive relationship between wall shear stress and pillar formation.

## Discussion

In this report, we studied the geometry of intraluminal tissue islands--here referred to as intravascular pillars--and their surrounding blood flow in the CAM. Intravital microscopy of vessel bifurcations demonstrated marked pillar anisotropy with pillars orienting along the streamlines of the dominant vessel. Mechanical forces potentially influencing pillar geometry were mapped using 3D computational simulations. Shear maps demonstrated that pillars were predictably constrained by regions of high wall shear stress. Further, the development of new pillars was limited to regions with low shear stress. We conclude that mechanical forces have both a limiting and permissive influence on pillar development in the CAM.

Defining the relationship between mechanical forces and blood flow is experimentally challenging. Focal structural changes can be difficult to recognize in 2D intravital imaging [33]. Definitive structure can be revealed by techniques such as corrosion casting and 3D scanning electron microscopy [39], but static imaging cannot provide a simultaneous assessment of blood flow. Global morphologic alterations, more readily identified by intravital microscopy, are confounded by the inter-relationship between blood flow and structure. In contrast, intravascular pillars provide a unique opportunity to assess both structure and blood flow. Pillars are discrete structures within the blood stream, relatively isolated from extravascular soluble tissue factors, that are not only identifiable by intravital 2D imaging, but amenable to detailed morphometric analysis. The pillars are lined with normal-appearing endothelium [23] suggesting a normal responsiveness to intraluminal flow fields.

Computational flow modeling provides important insights into flow-associated mechanical forces. The mechanical forces associated with blood flow include wall shear stress (the frictional force tangential to the vessel wall) and circumferential strain (a blood pressure-related force perpendicular to the direction of flow); wall shear stress being the dominant mechanical force in the smooth and continuous flow of the peripheral CAM microcirculation. Although wall shear stress cannot be directly measured, numerical simulations enable not only the calculation of wall shear stress, but the mapping of these mechanical forces to the vessel wall. The spatial relationships revealed by these maps provide important insights into the role of mechanical forces in shaping vessel structure and the contribution of mechanical forces in localized disease processes such as atherosclerosis.

Despite the value of a numerical analysis, there were also limitations of our computational approach. First, our computational simulation treated the blood as a Newtonian fluid and discounted the mechanical effects of blood cells. We have used a continuum approach in this study, because the chick chorioallantoic membrane has a blood cell concentration that is significantly lower, and more variable, than adults [40]. This observation suggests that the structural modification is relatively insensitive to blood cell concentration. An alternative to continuum modeling is a discretized approach such as discrete particle dynamics (DPD) [41-43]. We have successfully applied DPD to the analysis of blood components in a parallel-plate flow chamber [44]; however, the computational demands of this promising technique currently preclude its application to the complex geometry of the CAM. Despite the theoretical limitations of a continuum approach, we suspect that our computational model closely approximates the distribution of forces in vivo.

A second limitation of our computational approach is that we do not consider extravascular tissue resistance. Since tissue viscoelasticity can neither be derived from first principles nor measured in vivo, our simplifying assumption is that the vessels function as rigid tubes. Our rationale is that we are modeling smooth and continuous flow in the peripheral extra-embryonic vascular network; not the pulsatile flow in the peri-embryonic vessels. Furthermore, we are not attempting to define the overall energetics of the extra-embryonic tissues, but rather describe the spatial distribution of the mechanical forces within the microvessels. The value of defining force distribution is that it can provide not only a mechanical explanation for structural changes, but a predictive map of endothelial changes. We anticipate that shear maps will facilitate metabolic and transcriptional studies both in vitro (e.g. flow chambers) and in vivo (e.g. laser capture microdissection).

The spatial relationship of pillar anisotropy and wall shear stress suggests that blood flow shapes pillar geometry. The pillar axis was almost uniformly oriented along the streamlines of the dominant vessel in a bifurcation. Consistent with these findings, Thoma observed more than 100 years ago the importance of the "stromrichtung" in determining vessel structure [5]. The relevance of wall shear stress in influencing pillar structure is underscored by the absence of any comparable spatial relationship: the pillars were oriented randomly with respect to 1) different levels of the microcirculation, 2) the longitudinal axis of the embryo, 3) the vitelline (omphalomesenteric) vessels of the yolk sac and 4) the flow streamlines of neighboring nondominant vessels. The strong spatial coincidence of the streamlines and wall shear stress suggests that mechanical forces shape intravascular pillars in the CAM. We speculate that this observation may reflect a more general influence of mechanical forces on all microvessel endothelium.

The influence of blood flow on the lining of CAM microvessels is consistent with many observations of endothelial cells in vitro and intravascular pillars in vivo. The orientation and elongation of the intravascular pillars observed in our study is consistent with the sensitivity of endothelial cells to the direction of flow. Our observations are compatible with the cytoskeletal rearrangement, lamelipodial protrusion and mechanotaxis observed in culture [19-21]. The influence of intravascular pillars is also consistent with the branch angle remodeling and vascular pruning observed in vivo [45]. In each case, the elongation of the pillar likely reflects the dominant streamlines in the flow field. A notable exception is the observation of pillars in divergent flow streams [46]. In our study, new pillars were commonly observed at convergent flow streams or in locations "downstream" from pre-existing pillars. One explanation is simply sampling and/or technical limitations of our study. Another explanation is that pillars are typically formed at convergent bifurcations but are later observed at divergent flow streams after spontaneous flow reversal--an infrequent, but real, observation in the CAM microcirculation.

Because new pillars were not predictable from our shear maps, we suspect that mechanical forces have a permissive role in pillar development; that is, regions of low shear stress permit pillar development stimulated by other growth or developmental signals [47]. A diffusible endothelial activation signal provides one explanation for the vessel irregularities observed during pillar development. A competing hypothesis is that mechanical forces stimulate new pillar formation and may even initiate the related process of intussusceptive angiogenesis [48]. These are both intriguing and plausible possibilities that deserve further study.

## Abbreviations

2D: 2-dimensional; 3D: 3-dimensional; CAM: chorioallantoic membrane; EDD: embryonic development day; FEM: finite element model, FITC: fluorescein isothiocyanate

## Authors' contribution

All authors participated in design and conception of the study. GL was lead investigator in the intravital microscopy assisted by LM and ML. BG and DS contributed data analysis and study design. Flow dynamics simulations were performed by NF and AK. MK performed the corrosion casting and 3D scanning electron microscopy. SM contributed to study design and manuscript preparation. All authors have read and approved the manuscript.

## Competing interests

The authors declare that they have no competing interests.
